# Bimorph Dual-Electrode ScAlN PMUT with Two Terminal Connections

**DOI:** 10.3390/mi13122260

**Published:** 2022-12-19

**Authors:** Meilin Ji, Haolin Yang, Yongxin Zhou, Xueying Xiu, Haochen Lv, Songsong Zhang

**Affiliations:** 1 School of Microelectronics, Shanghai University, Shanghai 200444, China; 2 Shanghai Industrial µTechnology Research Institute, Shanghai 201800, China

**Keywords:** scandium-doped aluminum nitride (Sc_0.2_Al_0.8_N), bimorph, dual-electrode, PMUT

## Abstract

This paper presents a novel bimorph Piezoelectric Micromachined Ultrasonic Transducer (PMUT) fabricated with 8-inch standard CMOS-compatible processes. The bimorph structure consists of two layers of 20% scandium-doped aluminum nitride (Sc_0.2_Al_0.8_N) thin films, which are sandwiched among three molybdenum (Mo) layers. All three Mo layers are segmented to form the outer ring and inner plate electrodes. Both top and bottom electrodes on the outer ring are electrically linked to the center inner plate electrodes. Likewise, the top and bottom center plate electrodes are electrically connected to the outer ring in the same fashion. This electrical configuration maximizes the effective area of the given PMUT design and improves efficiency during the electromechanical coupling process. In addition, the proposed bimorph structure further simplifies the device’s electrical layout with only two-terminal connections as reported in many conventional unimorph PMUTs. The mechanical and acoustic measurements are conducted to verify the device’s performance improvement. The dynamic mechanical displacement and acoustic output under a low driving voltage (1 Vpp) are more than twice that reported from conventional unimorph devices with a similar resonant frequency. Moreover, the pulse-echo experiments indicate an improved receiving voltage of 10 mV in comparison with the unimorph counterpart (4.8 mV). The validation of device advancement in the electromechanical coupling effect by using highly doped ScAlN thin film, the realization of the proposed bimorph PMUT on an 8-inch wafer paves the path to production of next generation, high-performance piezoelectric MEMS.

## 1. Introduction

Piezoelectric Micromachined Ultrasonic Transducers (PMUTs) have attracted more attention to the continuously growing market demand in applications requiring miniaturized ultrasound technology. Compared with conventional ultrasonic transducers, PMUTs have advantages, such as small size, low power consumption, and cost-effective batch manufacturing [1]. PMUTs are used in a wide range of industrial, medical, and consumer electronics applications, such as rangefinders [2], medical ultrasound imaging [3], fingerprint recognition [4], and so on.

In recent years, MEMS ultrasonic transducers have been rapidly developed due to the progression in piezoelectric thin film technology. Lead zirconate titanate (PZT) has been investigated as a thin film piezoelectric material for PMUT because of its outstanding piezoelectricity [5]. However, during practical device integration, thin film PZT normally requires a higher processing temperature above 450 °C, which is not compatible with most CMOS processes [6]. Aluminum nitride thin film, on the other hand, can be deposited at a much lower temperature (≤300 °C) and has excellent electrical and thermal properties. But its piezoelectric coefficient is relatively small compared with PZT and leads to a low electromechanical coupling effect for an actuating purpose [7]. One way to overcome this problem is to use scandium-doped aluminum nitride (ScAlN) thin film. ScAlN has great potential in piezoelectric MEMS devices since it is a CMOS-compatible fabrication process and has a higher piezoelectric coefficient than aluminum nitride [8,9,10]. Qi Wang et al. successfully fabricated the PMUT on cavity-SOI wafers based on Sc_0.15_Al_0.85_N films. The device achieved a transmitting sensitivity of 22 nm/V at a resonant frequency of 17.5 MHz in air. Compared to the same size AlN PMUT, the transmitting sensitivity of Sc_0.15_Al_0.85_N PMUT is doubled [11].

In addition to film technology transformation, there are many other ways to improve the performance of PMUT. Sina Akhbari et al., investigated PMUT with self-curved diaphragms by the stress of silicon nitride and low-temperature oxide (LTO) films. The dynamic deflection of curved PMUT was higher than similar-size flat PMUT [12]. Tao Wang et al. reported a piston-like PMUT. With the help of etched holes in the film, the mode shape was changed from Gaussian to piston-like, which resulted in higher transmitted sound pressure and sensitivity [13]. In addition to the above method of modifying the elastic layer, replacing the elastic layer with a piezoelectrically active layer could theoretically double the electromechanical coupling coefficient of the PMUT. Sina Akhbari et al. proposed the concept and basic theory of dual-electrode bimorph (DEB) PMUT [14]. The DEB PMUT architecture was a great replacement for the existing PMUT design. Under the differential drive scheme, theoretical analysis and experimental verifications showed four times greater driving sensitivity, in contrast to that of a state-of-the-art PMUT with similar geometry and frequency. In 2015, Liang Lou et al. validated the bimorph structure PMUT with pure AlN thin film fabricated on an 8-inch wafer. The recorded dynamic deflection from the bimorph design was 77% higher than that of its unimorph counterpart [15].

**Figure 1:**
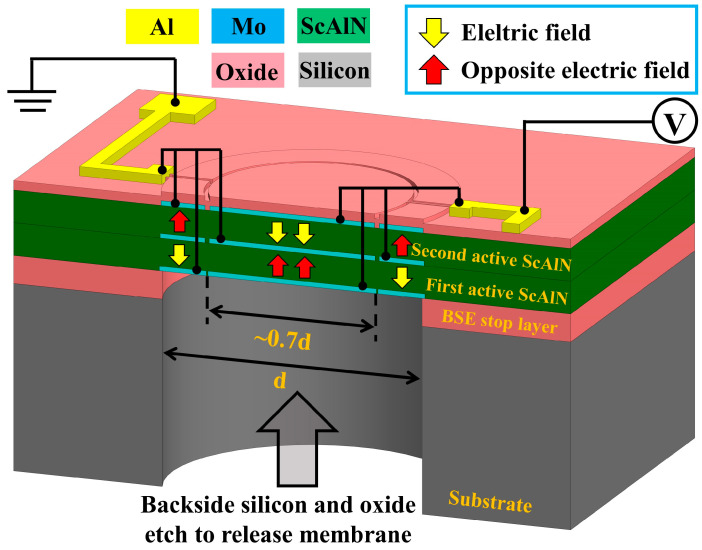
The schematic 3D cross-section drawing of the bimorph dual-electrode ScAlN PMUT.

In this paper, we present a bimorph dual-electrode Sc_0.2_Al_0.8_N PMUT with only two-terminal configuration. The 3D cross-section and electrical connection are shown in Figure 1. With the help of 20% doped ScAlN, the transmitting sensitivity is 2.38 times in comparison with AlN. Meanwhile, with the enlarged effective sensing area, the acoustic output is 2.42 times that of unimorph and the receiving sensitivity is 1.7 times. Compared with the unimorph PMUT, the overall pulse-echo receiving voltage has been significantly improved by two times.

## 2. Design and Simulation

We have verified the performance of the bimorph dual-electrode ScAlN PMUT using FEM simulations. The material parameters used in the FEM simulation are as shown in Table 1 [16].

**Figure 2:**
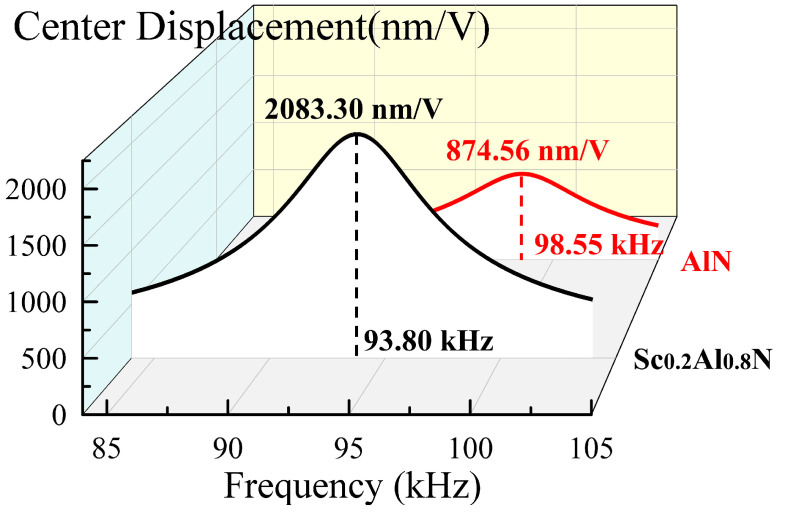
The FEM simulation dynamic mechanical amplitude for 500 μm diameter ScAlN and AlN bimorph PMUT.

Figure 2 compares the dynamic mechanical amplitude simulation results of ScAlN and AlN bimorph PMUT with 500 μm diameter. The ScAlN PMUT has 2.38 times the dynamic mechanical amplitude; as large as that of the AlN devices. The reason for this phenomenon is that for AlN films with wurtzite crystal structure, the piezoelectric response can be increased considerably by replacing the Al cation with an Sc cation [17]. On the other hand, the ScAlN PMUT has a lower resonant frequency than that AlN PMUT. The resonant frequency of circular PMUT is calculated as [11]:

(1) $$f=\frac{1.63}{r^{2}}\sqrt{\frac{D}{\sum \rho_{i}t_{i}}}$$

where f is the resonant frequency, D is rigidity, r is the PMUT radius, ρ and t are the density and thickness of each layer of the membrane. The rigidity D can be expressed as:

(2) $$D=\frac{1}{3}\overset{n}{\underset{i=1}{\sum }}\frac{E_{i}^{2}\left(z_{i}^{3}-z_{i-1}^{3}\right)}{1-v_{i}^{2}}$$

where $E_{i}$ is Young’s modulus, $v_{i}$ is the Poisson’s ratio of the material, $z_{i}$ is the distance of the i-th layer top surface from the neutral axis. Sc cations reduce the stiffness of AlN, which causes a lower resonance frequency of ScAlN PMUT than AlN PMUT as shown in Equation (1).

**Figure 3:**
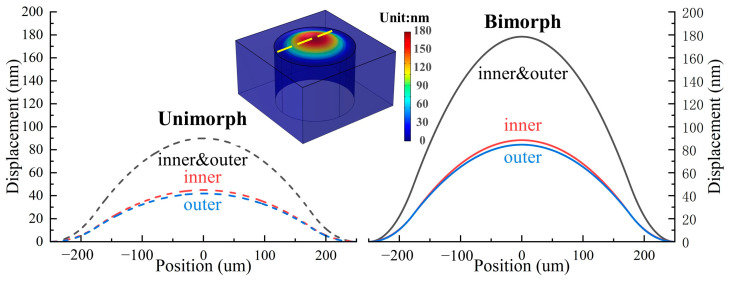
The FEM simulation static film displacement under 1 V_dc_ voltage for the bimorph and unimorph, dual-electrode and single-electrode.

Figure 3 shows the static film displacement under 1 V_dc_ voltage with four different applied voltages. The center displacement for a unimorph single-electrode PMUT (USP) driven by 1 V_dc_ is x_c_. The electrical and mechanical energy equations for the USP PMUT are [14]:

(3) $$E_{e\_USP}=\frac{1}{2}\left(\frac{0.5A_{p}\varepsilon_{p}}{h_{p}}\right)V_{dc}^{2}=\frac{1}{4}\left(\frac{A_{p}\varepsilon_{p}}{h_{p}}\right)V_{dc}^{2}$$

(4) $$E_{m\_USP}=\frac{1}{2}k_{mc}x_{c}^{2}$$

where the diaphragm area is $A_{p}$; the inner plate electrode and outer ring electrode areas are both equal to 0.5 $A_{p}$ (the inner area is slightly larger than the outer due to the presence of gaps between the inner plate and outer ring. The gap area is negligible); the piezoelectric layer thickness and permittivity are $h_{p}$ and $\varepsilon_{p}$, respectively; the effective mechanical stiffness at the center is $k_{mc}$. The efficiency during the electromechanical energy coupling process for USP is:

(5) $$\eta_{USP}=\frac{E_{m\_USP}}{E_{e\_USP}}=2\left(\frac{h_{p}k_{mc}}{A_{p}\varepsilon_{p}}\right){\left(\frac{x_{c}}{V_{dc}}\right)}^{2}$$

For the dual-electrode configuration, the inner plate electrode diameter is designed to be 70% of the PMUT diameter to attain the maximum electromechanical conversions [18]. The unimorph dual-electrode PMUT (UDP) electrode area is twice as large as that of the USP PMUT. Therefore, the input electrical energy is double that of a USP PMUT under the same input voltage. Meanwhile, the UDP center displacement is twice that of USP, and the output mechanical energy is four times higher, so the electromechanical efficiency is two times higher. For the UDP:

(6) $$E_{e\_UDP}=\frac{1}{2}\left(\frac{0.5A_{p}\varepsilon_{p}}{h_{p}}\right)V_{dc}^{2}+\frac{1}{2}\left(\frac{0.5A_{p}\varepsilon_{p}}{h_{p}}\right)V_{dc}^{2}=2E_{e\_USP}$$

(7) $$E_{m\_UDP}=\frac{1}{2}k_{mc}{\left(2x_{c}\right)}^{2}=4E_{m\_USP}$$

(8) $$\eta_{UDP}=\frac{E_{m\_UDP}}{E_{e\_UDP}}=4\left(\frac{h_{p}k_{mc}}{A_{p}\varepsilon_{p}}\right){\left(\frac{x_{c}}{V_{dc}}\right)}^{2}=2\eta_{USP}$$

The bimorph dual-electrode PMUT (BDP) center displacement and electrode area are four times that of USP. The energy equations are:

(9) $$E_{e\_BDP}=2\times \frac{1}{2}\left(\frac{0.5A_{p}\varepsilon_{p}}{h_{p}}\right)V_{dc}^{2}+2\times \frac{1}{2}\left(\frac{0.5A_{p}\varepsilon_{p}}{h_{p}}\right)V_{dc}^{2}=4E_{e\_USP}$$

(10) $$E_{m\_BDP}=\frac{1}{2}k_{mc}{\left(4x_{c}\right)}^{2}=16E_{m\_USP}$$

(11) $$\eta_{BDP}=\frac{E_{m\_BDP}}{E_{e\_BDP}}=8\left(\frac{h_{p}k_{mc}}{A_{p}\varepsilon_{p}}\right){\left(\frac{x_{c}}{V_{dc}}\right)}^{2}=4\eta_{USP}$$

**Figure 4:**
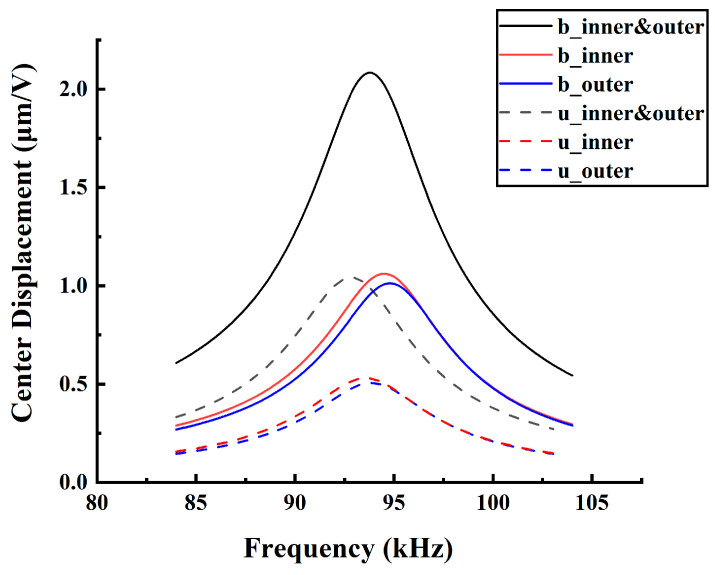
The FEM simulation dynamic sensitivity for the bimorph and unimorph, dual-electrode and single-electrode PMUT.

For BDP, the elastic layer is replaced by a piezoelectric layer, both ScAlN layers are activated and generate membrane deflection. None of the energy is used to deflect the elastic layer. Additionally, the dual-electrode electrical configuration maximizes the effective area of the given PMUT design. It generates higher displacement and output sound pressure. In summary, the efficiency of the electromechanical energy coupling process of the bimorph dual-electrode structure is four times higher than that of the unimorph single-electrode as shown in Figure 4.

We also optimized the electrical connection of BDE PMUT. Conventional PMUT with a dual-electrode usually requires a three-terminal configuration. It is necessary to apply reverse voltage to realize the differential drive of inner plate and outer ring electrodes [19]. However, the reverse voltage makes the transmitting circuit more complicated, especially for interconnection routing in PMUT array design [20]. Here, we propose bimorph dual-electrode PMUT with only two terminal connections. Both top and bottom electrodes on the outer ring are electrically linked to the center inner plate electrodes. Likewise, the top and bottom center plate electrodes are electrically connected to the outer ring in the same fashion as shown in Figure 1. The electrical link reduces terminal connections for a single PMUT and simplifies the electrical layout of interconnection for large PMUT arrays in the future.

## 3. Fabrication

### 3.1. Process Flow

**Figure 5:**
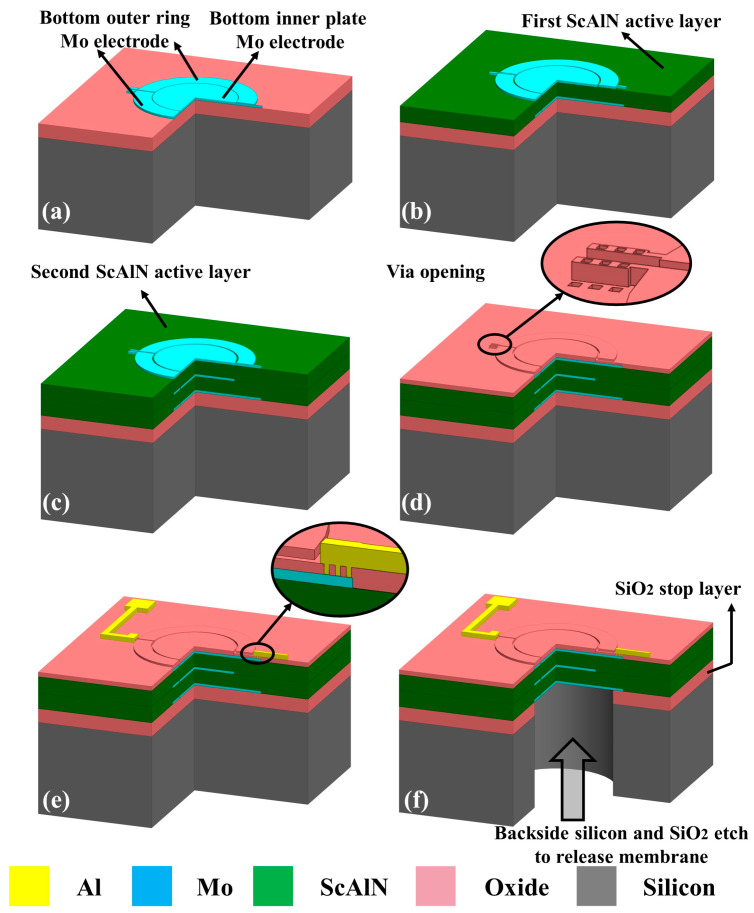
The illustration of the process flow ((**a**–**f**) in sequence) for the fabrication of bimorph dual-electrode ScAlN PMUT: (**a**) bottom Mo electrode sputtering and patterning; (**b**) first active ScAlN and middle Mo electrode sputtering, middle Mo electrode patterning; (**c**) second active ScAlN and top Mo electrode sputtering, top Mo electrode patterning; (**d**) PECVD SiO_2_ deposition and via etching; (**e**) metallization to complete the interconnection of PMUT; (**f**) backside silicon and SiO_2_ etch to release membrane.

The CMOS-compatible process flow of bimorph PMUT is shown in Figure 5. It is very similar to the conventional unimorph PMUT process and does not add any other complicating process. To start with, a bare wafer with 500 nm thick thermal oxide on the surface allows the thermal oxide layer to act as a stop layer for the backside Deep Reactive Ion Etching (DRIE). Firstly, a ScAlN/Mo stack with thicknesses of 50 nm, and 200 nm is used for sputtering. Pattered Mo is the bottom electrode. The 50 nm thickness ScAlN is a seed layer to achieve a good crystalline structure for the subsequent Mo and ScAlN layers (a). Secondly, sputtering of 600 nm thickness of the first ScAlN active layer and 150 nm thickness Mo. Pattering Mo is the middle electrode (b). Similarly, repeat the previous step to form a second ScAlN active layer and top Mo electrode (c). Mo electrodes and ScAlN films are deposited on the Sigma^®^ standard PVD process chamber (Sigma^®^ Deposition System from SPTS, New-port, UK) using dc magnetron sputtering. For ScAlN deposition, an Al-Sc target with a chemical composition of Sc_0.2_Al_0.8_ is sputtered in a high purity (99.9995%) Ar/N_2_ atmosphere. Metal electrode PVD utilizes Ar only. Substrate temperature largely influences the kinetic energy available to the atoms on the surface of the deposited films. This energy increases proportionally with temperature, which helps in depositing the highly c-axis-oriented films [21]. We deposit ScAlN on top of molybdenum (Mo). The sputtering temperature is at 300 °C. Figure 5d shows the 200 nm thickness PECVD SiO_2_ deposition and vias dry etching. The dry etching in this step must be time-controlled to ensure the Mo layer does not get etched. The inductively coupled plasma (ICP, Omega^®^ etch system from SPTS, Newport, UK) etching of ScAlN thin films is optimized by adding BCl_3_/Ar pretreatment to the Cl_2_/BCl_3_/Ar main etching scheme. The native ScAlN oxide can be effectively removed by a short exposure to BCl_3_/Ar plasma. Compared to the chlorine-based plasma etching, Cl_2_/BCl_3_/Ar is found to have the highest etch rate for both ScAlN and its native oxide [22]. On the other hand, the addition of BCl_3_ to the Cl_2_/Ar mixture helps to improve the surface smoothness of plasma etched ScAlN [23]. If the etched ScAlN surface shows substantial roughness. During over-etching, this roughness is transferred into a bottom Mo layer, where the contact of Al to a bottom Mo is fabricated after the opening of vias in ScAlN. Such contacts could be prone to poor reliability due to large surface roughness [24]. After vias etching, sputtering and patterning Al as terminal connections (e). The final step is the backside DRIE process to release the membrane (f).

### 3.2. Fabrication Results

**Figure 6:**
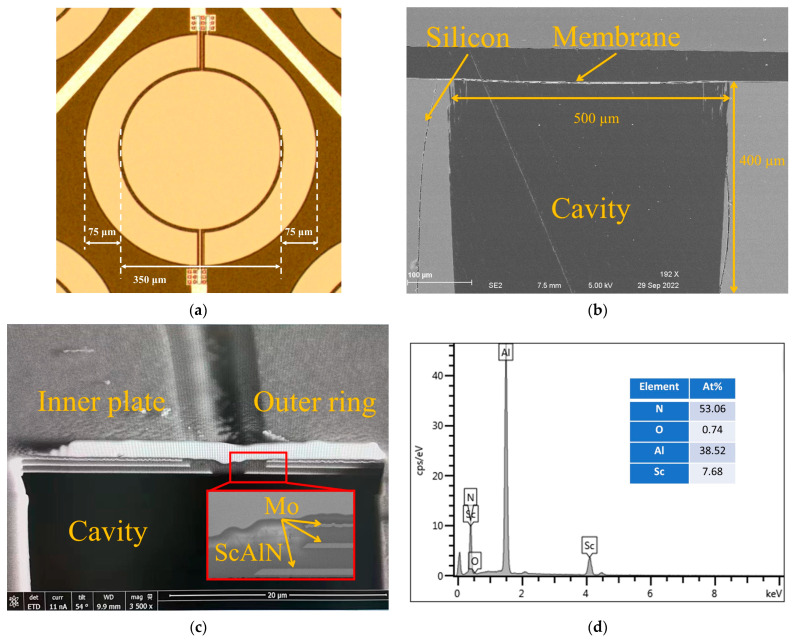
The fabrication results: (**a**) The optical microscope image of device top view; (**b**) The cross-section SEM image of bimorph dual-electrode ScAlN PMUT; (**c**) The bimorph films stack SEM image; (**d**) EDX results of Sc_0.2_Al_0.8_N films.

The fabrication results of bimorph dual-electrode ScAlN PMUT are shown next. Figure 6a is the optical microscope image of the device’s top view. It can be seen clearly that the electrodes are divided into inner plate and outer ring by etching. Figure 6b illustrates a cross-section SEM picture. The width of the cavity is 500 μm which defines the diameter of the PMUT. The depth of the cavity is 400 μm. SEM image of bimorph films stack after Focused Ion Beam (FIB) cutting are shown in Figure 6c. The abnormal protrusion on the top of the device is gold. Spraying gold is helpful to improve the SEM image quality. The inset shows the etching taper profile of the bottom electrode and middle Mo layer. To get improved characteristics of bimorph PMUT, it is essential to grow the high-quality ScAlN films and suppress the poor formation of ScAlN around the edge region of the mesa-shaped membrane. By optimizing the etching conditions of Mo, we could obtain the proper etching profile enough to get a well-grown ScAlN around the edge of the mesa structure. The proportion of aluminum and scandium is measured by energy-dispersive X-ray spectroscopy (EDX). The EDX results are shown in Figure 6d. The results show a consistent scandium concentration of ~20% throughout the film.

**Figure 7:**
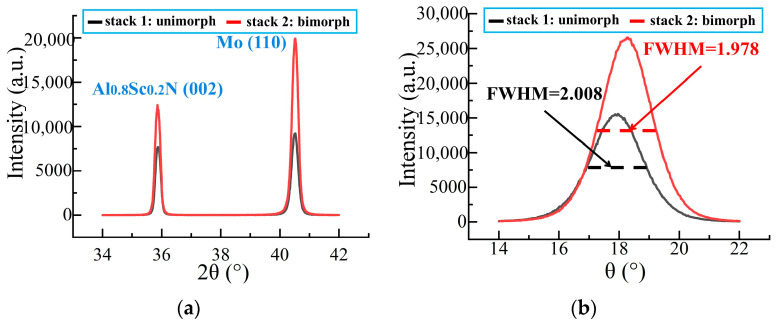
The fabrication results: (**a**) XRD measurement of the films stack; (**b**) Rocking curve measurement of ScAlN (002) peak.

The XRD and rocking curve measurement of two different film stacks: stack 1 (unimorph: ScAlN/Mo/ScAlN) and stack 2 (bimorph: ScAlN/Mo/ScAlN/Mo/ScAlN) are shown in Figure 7. It is found that bimorph stack in the (002) crystalline direction has a higher amplitude and slightly narrower FWHM as compared to the unimorph stack, indicating good quality of both the first and the second Sc_0.2_Al_0.8_N layers [25]. The unimorph film’s full-width-half-maximum (FWHM) of the (002) peak is 2.008° and the bimorph is 1.978°, which confirms that the film’s c-axis is well aligned and indicates good piezoelectric properties [26]. All fabrication results show that our prepared Sc_0.2_Al_0.8_N has good quality.

## 4. Experiment

### 4.1. Dynamic Mechanical Displacement

In our experiment, a unimorph PMUT (600 μm diameter) with a piezoelectric layer of 1 µm Sc_0.2_Al_0.8_N and an elastic layer of 5 µm Si is also fabricated as a control for the mechanical and acoustic tests. 

**Figure 8:**
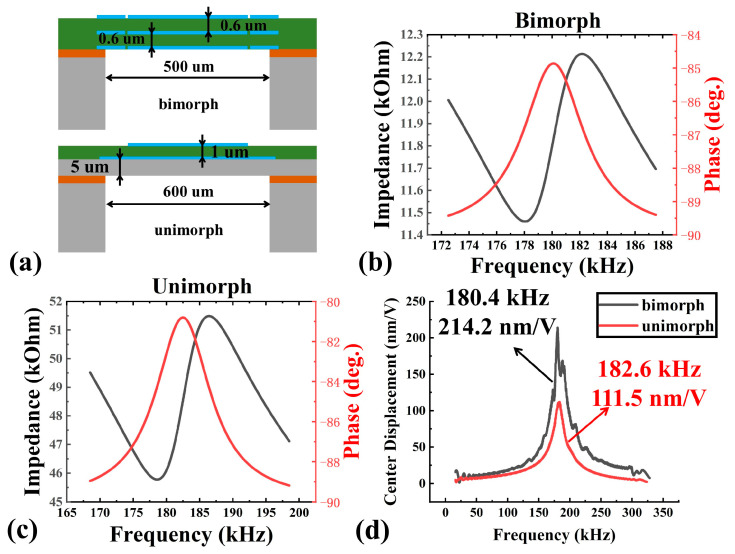
Device characterization for: (**a**) The schematic 2D cross-section drawing of bimorph and unimorph PMUT; (**b**) Electrical impedance characterization for bimorph; (**c**) Electrical impedance characterization for unimorph; (**d**) Dynamic mechanical amplitude characterization for bimorph and unimorph.

As shown in Figure 8. The frequency response of bimorph AlScN PMUT and unimorph PMUT with similar frequency are tested in the air using an impedance analyzer and a laser Doppler vibrometer. The bimorph shows a 214.2 nm/V dynamic mechanical amplitude at 180.4 kHz. The unimorph only shows a 115.5 nm/V dynamic mechanical amplitude at 182.6 kHz. This represents an improvement of 185%.

**Figure 9:**
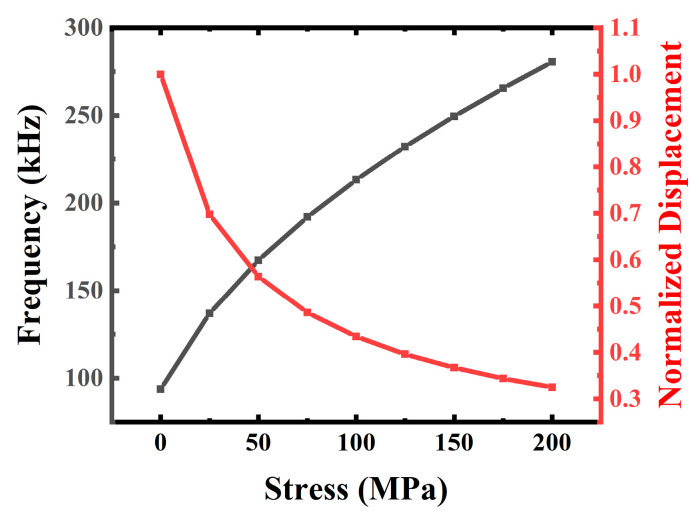
FEM simulation results of the normalized dynamic mechanical amplitude and resonant frequency for bimorph PMUT with different tensile residual stress.

However, the experimental results are lower in amplitude and higher in resonant frequency compared to the simulation results. The difference between simulation and measurement is caused by the overall membrane residual stress. As shown in Figure 9, with the residual tensile stress increasing, the frequency of the device increases and the dynamic mechanical displacement decreases. The PMUT resonant frequency is very sensitive to stress when the ratio of the radius to the thickness (r/t) is large [27]. The r/t of bimorph is larger than that of conventional unimorph devices on a silicon elastic layer, and residual stress control is a key issue to be studied in bimorph devices.

### 4.2. Transmitting Performance 

**Figure 10:**
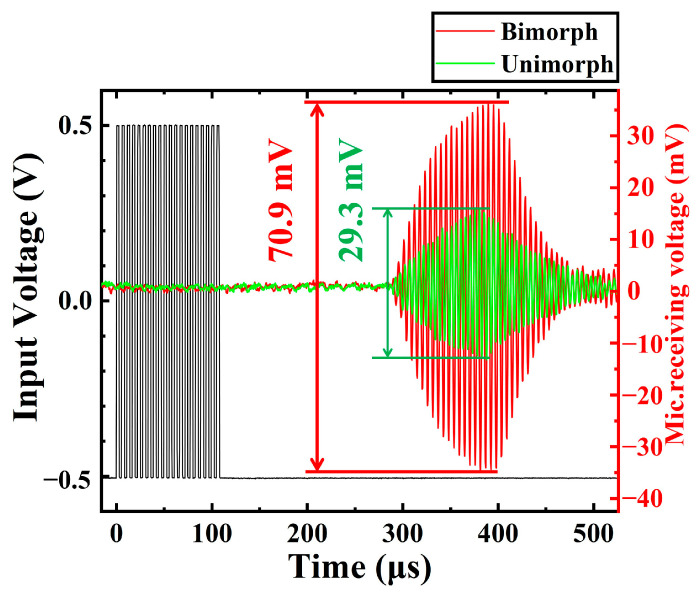
Device characterization for performance of transmitter.

**Figure 11:**
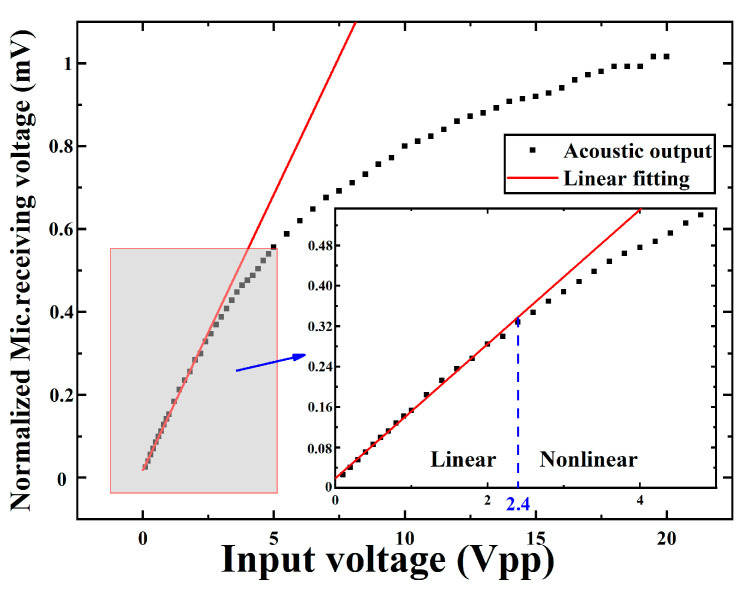
The acoustic output of PMUT under different voltages (0–20 V).

We compared the transmitting performance using a microphone (CM16/CMPA, 2kHz-200 kHz, Avisoft Bioacoustics Co. Ltd., Glienicke/Nordbahn, Germany). The microphone was placed 10 cm directly above the PMUT. Figure 10 shows the measurement compared results of the acoustic output between bimorph and unimorph. The single element of PMUT is excited by 20 cycles, 1 Vpp pulsed continuous wave. Compared to the unimorph, the acoustic output by the bimorph increased by 2.42 times. According to the microphone receiving voltage, the bimorph PMUT has greater transmitting performance. We also measure the acoustic output of PMUT under different voltages (0–20 V). As shown in Figure 11, when the applied voltage is 2.4 V, the device progressively enters from linear to non-linear region.

### 4.3. Receiving Performance

**Figure 12:**
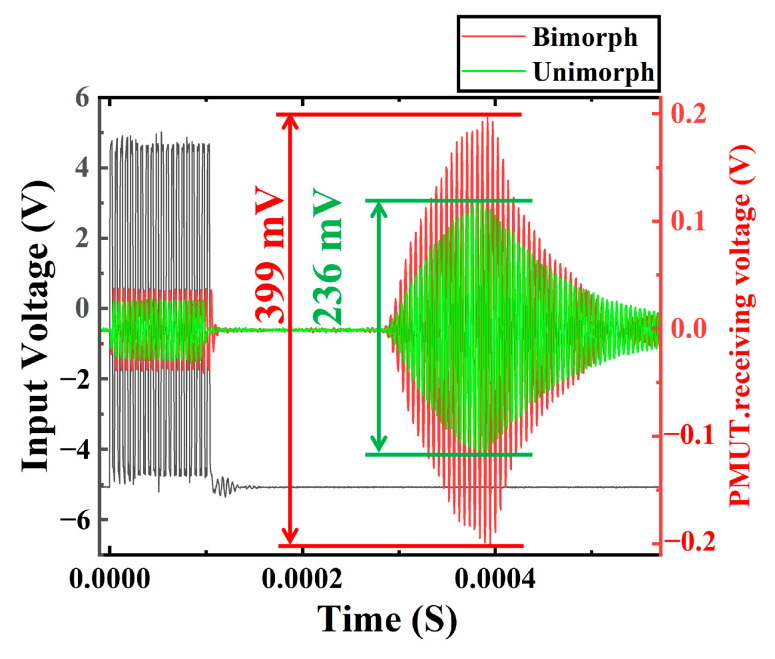
Device characterization for performance of receiver.

A commercial bulk ultrasonic transducer (HT0013, 190 ± 15 kHz, AUDIOWELL Co. Ltd., Guangdong, China) is used for the receiving performance experiment. An actuating voltage of 20 cycles, 10 Vpp was applied. The bulk ultrasonic transducer was also placed 10 cm directly above the PMUT. Figure 12 shows the compared measurement results of the PMUT receiving performance between bimorph and unimorph. Compared to the unimorph, the receiving performance by the bimorph increased by 1.7 times. 

### 4.4. Pulse-Echo Experiments

**Figure 13:**
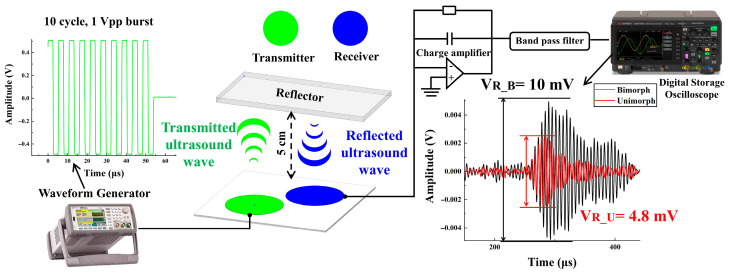
Setup and results for pulse-echo experiment.

Pulse-echo measurement is demonstrated, as shown in Figure 13. Two chips including bimorph and unimorph are placed on the same PCB, and a plastic plate reflector is placed 5 cm away from the chips. There are two membranes on each chip, one for transmitting and the other for receiving. An ultrasonic burst is transmitted by applying a 1 Vpp burst with ten cycles. The pulse-echo experiment of both chips is performed under identical experimental conditions (same burst, same charge amplifier, and same environment, etc.). The receiving voltage (V_R_) by the bimorph is 10 mV and the unimorph is 4.8 mV. Pulse-echo experiments demonstrate the better acoustic performance of the bimorph compared to the unimorph under low voltage (1 Vpp) drive. 

## 5. Conclusions

In this paper, we demonstrate the performance of the bimorph, dual-electrode PMUT based on a Sc_0.2_Al_0.8_N piezoelectric film. According to the FEM simulation results, using 20% Sc, the transmit sensitivity is increased by a factor of 2.38 relative to pure AlN PMUT. By using the bimorph dual-electrode structure to its advantage, the efficiency of the electromechanical energy coupling process is increased, as is the effective sensing area. The experimental results verify this conclusion. Compared with the unimorph single-electrode counterpart, the bimorph dual-electrode has 2.42 times higher acoustic output and 1.7 times higher receiving sensitivity. The pulse-echo receiving voltage by the bimorph is two times that of the unimorph. Both bimorph and unimorph pulse-echo experiments are performed with a low voltage (1 Vpp) drive. According to the results of the above experimental measurement, the bimorph has a small size and better acoustic performance than the unimorph over a similar frequency. This implies the bimorph dual-electrode ScAlN PMUT has great potential in the consumer electronics field.

## Figures and Tables

**Table 1:** The material parameters used in FEM simulations.

Material	E [Gpa]	ν	ρ [kg/m^3^]	e_31, f_ [C/m^2^]
AlN	338	0.30	3230	1.05
Sc_0.2_Al_0.8_N	230	0.23	3520	1.6

## Data Availability

The data are available within the article.
